# Prevalence of substance use disorders and associations with mindfulness, impulsive personality traits and psychopathological symptoms in a representative sample of adolescents in Germany

**DOI:** 10.1007/s00787-023-02173-0

**Published:** 2023-02-28

**Authors:** Nicolas Arnaud, Lutz Wartberg, Kathrin Simon-Kutscher, Rainer Thomasius, IMAC-Mind Consortium

**Affiliations:** 1 German Centre for Addiction Research in Childhood and Adolescence, University Medical Centre Hamburg-Eppendorf, Martinistr. 52, 20246 Hamburg, Germany; 2 Medical School Hamburg (MSH), Faculty of Human Sciences, Department of Psychology, University of Applied Sciences and Medical University, Am Kaiserkai 1, 20457 Hamburg, Germany

**Keywords:** Substance use disorders, Prevalence, Alcohol, Cannabis, Mindfulness, Adolescence

## Abstract

Adolescence is a critical phase for the development of substance use disorders (SUDs). For Europe and Germany, there are limited data on problematic substance use from representative youth samples. Trait mindfulness is relevant in buffering against substance use-related problems and associated deficits in self-regulatory control. The objective of this study is to estimate 12-month prevalence rates for SUDs in a representative sample of adolescents in Germany and to examine associations with mindfulness and related factors such as stress, impulsivity, sensation seeking and symptoms of psychopathology. A sample of 4001 adolescents aged 12–18 years from Germany was surveyed based on DSM-IV diagnostic criteria for SUDs. Logistic regression analyses were used to examine associations with mindfulness-related variables. Criteria of at least one of the assessed SUDs were endorsed by 11.2% of the adolescents. Alcohol use disorders had the highest prevalence rate (10.1%) followed by cannabis use disorders (2.6%). For both substances, the criteria for abuse were met about twice as often as those for dependence. The prevalence of cigarette dependence was 1.7%, while the prevalence for e-cigarette dependence was only 0.1%. Prevalence rates were higher for male youth and increasing with age. SUD prevalence was also related to mindfulness, impulsivity and sensation seeking and externalizing symptoms of psychopathology. The statistically significant associations varied across different SUDs. SUDs appear highly prevalent among German adolescents. The results have public health implications with regard to treatment needs and prevention measures in the youth population in Germany.

**Supplementary Information:**

The online version contains supplementary material available at 10.1007/s00787-023-02173-0.

## Introduction

Adolescence is a critical phase for the development of substance-related and other comorbid mental disorders [1, 2]. Substance use disorders (SUDs) such as abuse and dependence are related to the repeated intake of psychotropic substances such as alcohol, cannabis and other illegal drugs and are characterized by a person’s limited ability to control substance-related behavior despite the experience of significant negative consequences [3]. In Europe substance-related disorders are the most common reason for hospitalization in the adolescent and young adult population [4, 5].

In Germany, as in most European countries, nicotine and alcohol are the primary substances (mis-)used by teenagers as well as in the general population, while cannabis is the most widely used illegal substance [6]. Recently, 10.6% of German adolescents aged 12–17 years reported to have used illicit drugs at least once in their lives [7]. This value was almost completely attributable to cannabis (10.4%).

Current knowledge about prevalence rates of SUDs in Germany is restricted to the adult population. A recent population-based study reports the following 12-month prevalence rates for adults in Germany (18–64 years): 7.8% for nicotine dependence, 4.0 and 4.8% for abuse and dependence of alcohol, and 0.7 and 0.9% for abuse and dependence of cannabis, respectively [8]. Young adults (age 18–20) reported higher rates of dependence and abuse than any other age group in this survey. Although prevalence estimates for youth under the age of 18 years are important to determine needs for treatment [9], they are frequently inferred from the mid-nineteen-nineties EDSP study (Early Developmental Stages of Psychopathology [10]), a community-based sample of 3021 adolescents and emerging adults (14–24 years) from a southern German metropolitan area. In this sample, 4.9% of the adolescents and young adults met the criteria for abuse or dependence of any type of illicit drug (mainly cannabis: 4.2%) [11]. However, the main focus of the prospective cohort was to address etiological factors for psychopathology and the course of substance use and related disorders over time in a regional sample [10, 12].

Other sources of information include cross-sectional data from the United States: the National Survey on Drug Use and Health [13] reported a 12-month prevalence rate of 2.8% for alcohol abuse or dependence and 4.9% for drug abuse or dependence (mainly cannabis: 4.1%) in a subsample of 3936 adolescents aged 12–17 years. Results from the National Comorbidity Survey Replication Adolescent Supplement (NCS-A [14]) provide higher estimates of 12-month prevalence for substance abuse or dependence of 8.3% in a community-based sample of 10,123 adolescents aged 13–17 years living in the USA. These results suggest that SUDs might be highly prevalent in this age group in Western industrialized societies.

A multitude of interrelated biological, psychological and social factors contribute to the development of SUDs [15, 16]. From a neurobehavioral perspective, individual susceptibility for substance misuse and other risk-taking behavior during adolescence is (in part) explained by a developmental “imbalance” in the neurocircuitry underlying self-control. In this view, the maturation of the prefrontal cortex, which is crucial for cognitive control and executive functions that are involved to resist reward-related temptations, continues long into adulthood. Subcortical networks involved in affective processing, reward learning and impulsive behaviors, however, are developed early in life [16, 17]. Previous research indicates that impulsivity and sensation seeking can be considered as two independent and relevant personality risk factors for problematic substance use among adolescents [18, 19].

Mindfulness is commonly conceptualized within a framework of self-control and involves full attention to present-moment experience with an attitude of acceptance, non-judgment and openness [20–22]. Research indicates that mindfulness is related to self-regulatory control of behavioral impulses with a growing literature addressing the implications in the context of addictive behavior [23, 24] and other externalizing and internalizing psychological problems [25, 26]. Mindfulness is a growing theme in contemporary health psychology and addiction research and plays a protective role for problematic substance use [20–24]. Research also suggests that mindfulness can be measured as an individual characteristic (trait mindfulness), which refers to a person’s tendency to act attentive and non-judgmental to everyday-life experiences [27, 28].

In the current study, we examined the 12-month prevalence of SUDs for alcohol, cigarettes/ e-cigarettes and cannabis in a representative sample of German youth. We also examined associations between trait mindfulness, impulsive traits (impulsivity and sensation seeking) and psychopathological symptoms with the different SUDs.

## Methods

### Procedures and participants

This population-representative cross-sectional study is part of a larger collaborative research initiative with a focus on substance-related addictive disorders in Germany (*IMAC-Mind: Improving Mental Health and Reducing Addiction in Childhood and Adolescence through Mindfulness: Mechanisms, Prevention and Treatment*). It focuses on the clinical utility of mindfulness-oriented approaches to prevention and treatment of SUDs among children and adolescents. One aim of the research initiative is the estimation of clinical epidemiology of SUDs in Germany and to examine associations with mindfulness-related factors (see www.imac-mind.de [29]).

The data collection took place from October to November 2020 using computer-assisted telephone interviewing. It was done by a survey research institute (*forsa. Gesellschaft für Sozialforschung und statistische Analysen mbH*) experienced in conducting large-scale survey studies such as the national monitoring of substance use behavior of young people [7]. The interviewers were trained specifically for the present study. A final sample of 4001 adolescents was selected from an initial stratified probability sample of eligible adolescents in a multi-stage random procedure, designed to be representative of the national population of the targeted age group (a flowchart of data collection is provided in the supplement). The average time required to conduct the telephone interview and collect the data was 18.2 min. Individual consent to participate in the survey was obtained by telephone from the respective interviewer before the interview started. For 12- and 13-year-olds, the consent of their legal guardians was also obtained. A total of 10 contact attempts were made to reach the selected household or respondent. The data were weighted to adjust for differential probabilities of selection, differential nonresponse, and deviations in sociodemographic and geographic variables (i.e., federal state, community size, sex/gender, and birth year) between the sample and recent national census data (Microcensus 2017 of the Federal Statistical Office of Germany). The applied weighting scheme conforms common standard in survey studies; a detailed description about weighting can be obtained on request. Recruitment, consent, and field procedures were approved by the local ethics committee of the University Medical Centre Hamburg-Eppendorf (LPEK-0196).

## Measures

### Assessment of substance use and substance use disorders

Substance use was assessed for cigarettes/e-cigarettes, alcohol and cannabis using the European School Survey Project on Alcohol and Other Drugs (ESPAD) format including lifetime, past 12-months and past 30 days use [6]. E-cigarettes were broadly defined to include any form of electronic cigarettes with no differentiation between specific products such as tobacco heaters or other products with or without nicotine. In case participants reported lifetime use of the assessed substances, presence of symptoms for substance abuse and dependence were assessed for each substance with a yes/no response option. The questions were designed to represent the SUD criterion symptoms according to the *Diagnostic and Statistical Manual of Mental Disorders–Fourth Edition* (DSM-IV) [30]. Because use of illegal drugs other than cannabis is infrequent among children and adolescents in Germany [7] and to reduce survey length, only cannabis was included in the survey.

DSM-IV specifies categorically distinct diagnoses for substance abuse and dependence, which have been used in survey research in and outside Germany [14, 31, 32]. According to this classification, substance dependence was defined as meeting the threshold for three of the seven dependence criteria within a 12-month period. Abuse was defined as meeting the threshold for one of the four abuse criteria, but not meeting the criteria for dependence. A person who met both abuse and dependence criteria for a particular substance was scored only as substance dependent. With regard to nicotine/tobacco, DSM-IV does not specify abuse but only dependence. For this study the criteria for nicotine dependence were applied to e-cigarettes, although e-cigarette dependence is not specified in the DSM-IV. The DSM-IV criteria for abuse and dependence are presented in the supplement material (1a and b).

### Explanatory variables

#### Psychopathological symptoms

The assessment of psychopathological symptoms was done using the Strengths and Difficulties Questionnaire (SDQ) [33, 34]. It provides a dimensional self-report measure of social, emotional and behavioral functioning over the last six months and has a 3-point response format (0 = “*not true*”, 1 = “*somewhat true*”, 2 = “*certainly true*”). Five scales are computed, each consisting of five items: ”emotional symptoms”, “conduct problems”, “hyperactivity/inattention”, “peer (relationship) problems”, and “prosocial behavior”. High scores indicate more psychopathological problems, whereas a high value regarding “prosocial behavior” is appraised as strength. For the present sample internal consistency for subscales was calculated using McDonald’s omega (*ω*) as it provides a more accurate measure than the more widely used Cronbach’s alpha [35]. The values for the subscales ranged from *ω* = 0.45 to *ω* = 0.72, indicating an unacceptable level of internal consistency for conduct problems (*ω* = 0.45), peer problems (*ω* = 0.45) and prosocial behavior (*ω* = 0.50), which is similar to previous epidemiological studies in Germany [36, 37]. Only the SDQ-subscales of emotional symptoms (*ω* = 0.72) and hyperactivity/inattention (*ω* = 0.68) reached a sufficient level of reliability and were included as indicators of internalizing and externalizing problems for further analyses.

#### Psychological stress

The Perceived Stress Scale (PSS-4) was included as a measure of psychological stress in the present survey, mainly because the data was collected during the COVID-19 pandemic. Although this instrument is not a measure for COVID-specific stress, the brief 4-item self-report scale has been shown in a previous study to predict COVID-related increases in psychological stress among youth in Germany [38]. Participants rated how frequently they have appraised their life as unpredictable, uncontrollable and overloading within the previous month. The 5-point scale responses (0 = “never”, to 4 = “very often”) were summed up to a score of 0–16, with higher scores indicating higher levels of perceived stress (*ω* = 0.58).

#### Impulsivity and sensation seeking

Given the survey design and requirements to limit survey length, we used highly abbreviated versions of the subscales impulsivity and sensation seeking from the Substance Use Risk Profile Scale (SURPS); 2 items per subscale (5-point response format (1 = “*do not agree*”, to 5 = “*totally agree*”)) were selected based on high factor loadings from previous studies [39, 40]. Higher mean scores indicate higher levels of impulsivity or sensation seeking.

#### Trait mindfulness

The Mindful Attention and Awareness Scale for Adolescents (MAAS-A) [41, 42]) was used to assess trait mindfulness. The self-report scale consists of 15 items using a 6-point response format (1 = “*almost always*”, to 6 = “*almost never*”). Ratings were combined into a single score with a high scores indicating high expression of trait mindfulness (*ω* = 0.81).

#### Sociodemographic characteristics

Data on participant sex/gender, age, place of residence, educational (i.e., school attendance), first- and second-generation migration background (i.e., person him- or herself or at least one parent was born outside of Germany) were assessed (see Table 1).

**Table 1:** Demographic characteristics of study participants (*N* = 4001)

Variable	Total sample (*N* = 4001)	Participants with SUD (*N* = 449)	Participants without SUD (*N* = 3551)	*P*^c^
	% (SE)	% (SE)	% (SE)	
Sex/gender				0.024^d^
Male	50.9 (0.1)	55.9 (0.1)	50.2 (0.1)	
Female	49.1 (0.1)	44.1 (0.1)	49.7 (0.1)	
Diverse	< 0.1 (0.1)	0 (0.1)	< 0.1 (0.1)	
Age, years				< 0.001^e^
12–13	31.7 (0.2)	1.8 (0.1)	35.5 (0.1)	
14–15	34.4 (0.2)	20.5 (0.1)	36.2 (0.1)	
16–18	33.9 (0.2)	77.7 (0.1)	28.3 (0.1)	
Migration background^a^				0.293
Yes	19.2 (0.1)	10.2 (0.1)	19.4 (0.1)	
School type^b^				0.950
Gymnasium	54.6 (0.4)	54.8 (0.2)	54.6 (0.1)	
Other	44.9 (0.4)	45.2 (0.2)	36.5 (0.1)	
Urbanicity (inhabitants)				0.736
< 5000	17.9 (0.1)	16.7 (0.1)	18.0 (0.1)	
> 5000–20,000	28.6 (0.1)	28.1 (0.1)	28.6 (0.1)	
> 20,000–100,000	26.7 (0.1)	28.5 (0.1)	26.5 (0.1)	
> 100,000–500,000	12.7 (0.1)	13.8 (0.1)	12.6 (0.1)	
> 500,000	14.1 (0.1)	12.9 (0.1)	14.2 (0.1)	
Trait mindfulness M (SD)	4.49 (0.71)	4.23 (0.70)	4.53 (0.70)	< 0.001
Impulsivity M (SD)	2.10 (0.67)	2.26 (0.67)	2.08 (0.67)	< 0.001
Sensation seeking M (SD)	2.80 (0.82)	3.08 (0.81)	2.77 (0.82)	< 0.001
Emotional symptoms M (SD)	2.73 (2.20)	3.31 (2.32)	2.66 (2.17)	< 0.001
Hyperactivity/inattention M (SD)	3.50 (2.08)	4.10 (2.10)	3.42 (2.07)	< 0.001

^a^Person or at least one parent not born in Germany

^b^“Gymnasium” is the highest educational level; other school types include “Realschule” (middle educational level, 17.3% of total sample), “Hauptschule” (low educational level, 3.3%) and vocational schools (4.8%), non-specified other school types (16.2%); participants who finished school (3.3%). No significant differences in SUD prevalence rates were found between different school types. Participants with SUD refers to participants who met the criteria for at least one of the assessed substance use disorders

^c^Results of *Χ*^2^–tests for categorical and *t* tests for continuous measures

^d^For comparison between male and female

^e^For comparisons between all three age groups

## Data analysis

The complete sample could be used for analyses. To estimate 12-month prevalence of SUD, relative frequencies were calculated with 95% confidence intervals (95% CI), both for the total sample and stratified by sex/gender and different age groups. Prevalence values for specific SUDs (dependence for cigarettes or e-cigarettes, abuse and dependence of alcohol and cannabis) were also calculated for the subsample of those respondents who reported actual use of the specific substance within the past 12 months. Prevalence rates of substance use (lifetime, 12 months and 30 days) were compared between different age groups, sex/gender and migration status using chi-square (*Χ*^2^) tests.

Multivariate logistic regression analyses were then used to identify associations between explanatory and response variables (Unspecified SUD, Multiple SUDs and SUD subtypes present vs. not present). Explanatory variables were sex/gender, age, migration status, sum scores of hyperactivity and emotional symptoms, mean scores of impulsivity, sensation seeking, and trait mindfulness as well as a sumscore for psychological stress (see supplement material 3 for intercorrelations of study variables). For all models, odds ratios (ORs) with 95% confidence intervals (CIs), *P* values (alpha-level set at 0.05) and Nagelkerkes *R*^2^ (based on multivariate model using forced entry method for all explanatory variables) are reported. All calculations were based on the weighted data set and were completed using the statistical software package SPSS version 27 (IBM Inc., Armonk, NY, USA).

## Results

### Prevalence of substance use

Consumption prevalence (lifetime, 12 months, 30 days) for the various substances by sample characteristics are presented in in the supplement material (Table 2). All types of substances were used more among males than females and sex/gender differences were apparent even among the youngest portion of the sample (youth ages 12–13 years). Taken together, the substance use reported here largely matches with existing survey data in this age group in Germany [7].

**Table 2:** Prevalence of substance use disorders by sample characteristics (weighted sample, *N* = 4001)

Variable	*n* (%)	Cigarette dependence	e-cigarette dependence	Alcohol abuse^a^	Alcohol dependence	Cannabis abuse^a^	Cannabis dependence	Multiple SUDs^d^	Unspecified SUD^c^	Group comparison
		*n* (%)	*n* (%)	*n* (%)	*n* (%)	*n* (%)	*n* (%)	*n* (%)	*n* (%)	*p* value^b^
Total	4001 (100)	69 (1.7)	6 (0.1)	281 (7.0)	123 (3.1)	74 (1.8)	31 (0.8)	152 (3.8)	449 (11.2)	–
Age Group										
12–13 years	1268 (31.7)	2 (0.6)	1 (0.1)	6 (0.5)	1 (0.1)	1 (0.1)	0 (0.0)	2 (0.2)	8 (0.6)	< 0.001
14–15 years	1377 (34.4)	10 (0.7)	3 (0.2)	66 (4.8)	19 (1.4)	9 (0.7)	6 (0.4)	22 (1.6)	92 (6.7)	
16–18 years	1356 (33.9)	57 (4.2)	2 (0.1)	209 (15.4)	103 (7.6)	64 (4.7)	25 (1.8)	128 (9.4)	349 (25.7)	
Sex/gender										
Female	1964 (49.1)	27 (1.4)	1 (0.1)	118 (6.0)	60 (3.1)	29 (1.5)	12 (0.6)	63 (3.2)	198 (10.1)	0.024
Male	2035 (50.9)	42 (2.1)	5 (0.2)	163 (8.0)	63 (3.1)	45 (2.2)	19 (0.9)	89(4.4)	251 (12.3)	
Migration										
Yes	768 (19.2)	17 (2.2)	2 (0.3)	53 (6.9)	14 (1.8)	15 (2.0)	5 (0.7)	29 (3.8)	78 (10.2)	0.310
No	3230 (80.7)	51 (1.6)	4 (0.1)	225 (7.0)	107 (3.3)	57 (1.8)	25 (0.8)	123 (3.8)	366 (11.4)	

This table depicts prevalence estimates according to DSM-IV (at least 1 out of 4 criteria for Substance Abuse; at least 3 out of 7 criteria for Substance Dependence)

^a^Prevalence rates for abuse refer to abuse without dependence (i.e., persons who met criteria for both disorders were counted for dependence but not included in the prevalence rate for abuse; prevalence rates were 8.9% (10.0% for male and 7.7% for female youth) for alcohol abuse with or without dependence and 2.5% (2.9% for male and 2.0 for female youth) for cannabis abuse with or without dependence). All SUD subtypes were more prevalent among males than females except for alcohol dependence. Rates of SUD prevalence differed across age groups, but not by other sociodemographic characteristics

^b^*p* value based on Chi-square test refers to differences in prevalence in Unspecified SUD by sociodemographic factors

^c^Unspecified SUD refers to participants who met the criteria for at least one of the assessed substance use disorders

^d^Multiple SUDs refers to the rate of persons who endorsed the criteria for at least two different SUDs

### Prevalence of substance use disorders

The percentage of participants who met the diagnostic criteria for any of the assessed SUDs was 11.2%, a proportion of 3.8% in the sample met the criteria for at least two different SUDs. The highest prevalence rate was found for alcohol-related disorders with a sample proportion of 7.0% for abuse and 3.1% for dependence. The prevalence rates for cannabis use disorders were smaller: 1.8% of the participants met the criteria for abuse and 0.8% those of dependence. Regarding the prevalence rates for cigarette (1.7%) and e-cigarette (0.1%) dependence there was a clear preponderance for tobacco versus electronic cigarettes.

All SUDs except alcohol dependence were more prevalent among males compared to females (*p* = 0.024 for unspecified SUD) and differences in prevalence were increasing with age groups (*p* < 0.001 for unspecified SUD). Prevalence rates also differed by migration status for cigarette and e-cigarette dependence (migration background was associated with a higher prevalence for these disorders) and alcohol abuse (migration background was associated with a lower prevalence) but not for any other outcome (see Table 2).

When the prevalence rates were calculated only for those respondents who reported actual use of the particular substance in the past 12 months (see Table 3), the percentages increased substantially for all substances. Notably, while in the total sample, the prevalence was highest for alcohol-related disorders, among the current users, this peak shifted to cannabis-related outcomes with a subsample prevalence of 28.0 and 11.7%, respectively, for cannabis abuse and dependence as compared to alcohol (17.7% abuse and 7.7% dependence). A striking difference in the extent of dependence was found for users of cigarettes vs e-cigarettes: current users of tobacco cigarettes met the diagnostic criteria for dependence almost eight times more often compared to e-cigarette users (18 vs. 2.4%).

**Table 3:** Prevalence of substance use disorders for participants who reported use of the substance during the past 12 months

Variable	Cigarette dependence	e-cigarette dependence	Alcohol abuse	Alcohol dependence	Cannabis abuse	Cannabis dependence
12 months use of substance	Tobacco/cigarettes	e-cigarettes	Alcohol		Cannabis	
	*n* = 383 (9.6%)	*n* = 243 (6.1%)	*n* = 1592 (39.8%)		*n* = 264 (6.6%)	
Total	69 (18.0)	6 (2.4)	281 (17.7)	123 (7.7)	74 (28.0)	31 (11.7)
Age group						
12–13 years	2 (15.4)	1 (7.1)	6 (13.6)	1 (2.3)	1 (100)	0 (0)
14–15 years	10 (12.3)	3 (4.0)	66 (13.6)	19 (3.9)	9 (25.0)	6 (16.7)
16–18 years	57 (19.7)	2 (1.3)	209 (19.7)	103 (9.7)	64 (28.2)	25 (11.0)
Sex/gender						
Female	27 (15.5)	1 (1.0)	118 (15.1)	60 (7.7)	29 (25.2)	12 (10.4)
Male	42 (20.1)	5 (3.3)	163 (20.1)	63 (7.8)	45 (30.2)	19 (12.8)
Migration						
Yes	17 (24.3)	2 (4.4)	53 (23.0)	14 (6.1)	15 (34.1)	5 (11.4)
No	51 (16.6)	4 (2.0)	225 (16.6)	107 (7.9)	57 (26.3)	25 (11.5)

This table depicts prevalence estimates according to DSM-IV (at least 1 out of 4 criteria for substance abuse; at least 3 out of 7 criteria for substance dependence) among those respondents who reported use of the specific substance in the past 12-months. Prevalence rates for abuse refer to abuse without dependence (i.e., persons who met criteria for both disorders were counted for dependence but not included in the prevalence rate for abuse; prevalence rates were 22.4% (25.2% for male and 19.5% for female youth) for alcohol abuse and 37.9% (40.3% for male and 34.8 for female youth) for cannabis abuse with or without dependence)

### Substance use disorders and associated factors

#### Unspecific SUD and multiple SUDs

The multivariable associations between explanatory variables and SUD-related outcomes for all calculated models are presented in Table 4. In this analysis, male sex/gender, older age, higher scores on SDQ-dimensions for emotional problems and hyperactivity symptoms, lower scores trait measures of mindfulness, and higher scores on sensation seeking and impulsivity were associated with statistically significant increased risk for meeting DSM-IV criteria for any of the assessed SUDs. A similar pattern of associations was also found with the occurrence of multiple (i.e., > 1) substance use disorders, with the difference that trait mindfulness was not a statistically significant factor in this model.

**Table 4:** Sociodemographic and psychosocial factors and associated likelihood with fulfillment of diagnostic criteria for DSM-IV substance use disorder (*N* = 3999)

	Unspecified SUD	Multiple SUDs	Cigarette dependence	Alcohol abuse	Alcohol dependence	Cannabis abuse	Cannabis dependence
Sex/gender	1.58*** **[1.24, 2.02]**	2.02*** **[1.35, 3.02]**	2.60*** **[1.45, 4.65]**	1.66*** **[1.90, 2.16]**	1.21 [0.80, 1.85]	1.88** **[1.17, 3.02]**	3.53** **[1.15, 8.46]**
Age	2.27*** **[2.07, 2.48]**	2.26*** **[1.95, 2.62]**	2.20*** **[1.78, 2.71]**	2.09*** **[1.90, 2.29]**	2.23*** **[1.90, 2.63]**	2.26*** **[1.89, 2.70]**	2.00*** **[1.49, 2.71]**
Migration	0.87 [0.65, 1.16]	0.97 [0.62, 1.50]	1.35 [0.75, 2.43]	0.92 [0.68, 1.25]	0.15* **[0.29, 0.92]**	1.01 [0.58, 1.72]	0.64 [0.24, 1.76]
Stress (PSS-4)	1.00 [0.95, 1.05]	1.04 [0.97, 1.12]	1.01 [0.91, 1.13]	1.03 [0.98, 1.09]	0.95 [0.87, 1.03]	1.00 [0.92,1.11]	1.2* **[1.03, 1.41]**
Emotional symptoms (SDQ)	1.07* **[1.00, 1.13]**	1.13* **[1.02, 1.24]**	1.22** **[1.06, 1.39]**	1.06 [0.99, 1.13]	1.06 [0.95, 1.18]	1.11 [0.99, 1.25]	1.24* **[1.02, 1.41]**
Hyperactivity-inattention (SDQ)	1.15** **[1.08, 1.22]**	1.23*** **[1.15, 1.38]**	1.24*** **[1.09, 1.42]**	1.15*** **[1.08, 1.22]**	1.08 [0.98, 1.20]	1.26*** **[1.13, 1.40]**	1.27* **[1.05,1.54]**
Trait mindfulness (MAAS)	0.78** **[0.64, 0.95]**	0.81 [0.59, 1.10]	0.82 [0.53, 1.27]	0.86 [0.69, 1.06]	0.54*** **[0.39, 0.77]**	0.85 [0.58, 1.22]	0.84 [0.44, 1.59]
Sensation seeking (SURPS)	1.50*** **[1.31, 1.73]**	1.58*** **[1.26, 1.99]**	1.35 [0.98, 1.85]	1.62*** **[1.38, 1.89]**	1.43** **[1.12, 1.83]**	1.34* **[1.03, 1.75]**	1.16 [0.74, 1.81]
Impulsivity (SURPS)	1.30** **[1.09, 1.57]**	1.46** **[1.11, 1.93]**	1.81** **[1.22, 2.68]**	1.26* **[1.04, 1.54]**	1.61** **[1.12, 2.19]**	1.03 [0.74, 1.44]	0.96 [0.53, 1.73]
Nagelkerkes *R*^2^	0.291	0.260	0.235	0.247	0.214	0.203	0.223

This table depicts the association between DSM-IV substance use disorders (any SUD and subtypes). Bold value indicates that odds ratios are statistically significant (*p < 0.05; **p < 0.01; ***p < 0.001). Adjusted Odds Ratios (aORs) and 95% Confidence Intervals (95%-CIs) and Nagelkerkes *R*^2^ are based on multivariable logistic regression models using forced entry method. Sex/gender coding: 1 = male, 0 = female; Migration coding: 0 = no, 1 = yes. Age in years (12–18)

#### SUD subtypes: alcohol abuse and dependence

For alcohol abuse and dependence, the associations were similar in magnitude and significant for older age, sensation seeking and impulsivity. While alcohol abuse was significantly associated with male sex/gender, there was no such association for alcohol dependence. Alcohol dependence in turn was associated with migration; those adolescents who reported a migration background were significantly less likely to meet the criteria for alcohol dependence.

There were no statistically significant associations for SDQ-subscales of emotional problems and hyperactivity symptoms with alcohol dependence; however, higher levels of SDQ hyperactivity symptoms were associated with alcohol abuse.

Relations varied also for mindfulness: while participants with a higher score on mindfulness were significantly less likely to meet criteria for dependence, the odds for abuse fell just short of statistical significance. Higher scores for impulsivity and sensation seeking were significantly related to alcohol dependence and alcohol abuse.

#### SUD subtypes: cannabis abuse and dependence

For cannabis abuse, there was a very similar pattern of associations compared to alcohol abuse: male sex/gender, older age, SDQ hyperactivity symptoms and sensation seeking were positively and statistically significant associated with cannabis abuse, while impulsivity was not. For cannabis dependence the association with sex/gender was particularly strong: male youth were 3.5 times more likely to meet criteria for cannabis dependence than females. Furthermore, older age, SDQ-based higher levels of hyperactivity symptoms and emotional problems were significant factors; whereas, trait mindfulness, impulsivity and sensation seeking were not associated with cannabis dependence.

#### SUD subtypes: cigarette and e-cigarette dependence

Due to very low prevalence rates for e-cigarette dependence in the sample the requirements for statistical analysis were not met and associations were only calculated for tobacco cigarettes. For this outcome the associations were significant for age and sex/gender: older age was associated with higher risk for cigarette dependence and male youth were about 2.6 times more likely to meet criteria for this disorder. Other factors associated with cigarette dependence were emotional problems and hyperactivity symptoms, as well as impulsivity.

#### Additional analyses

There were notable (but not excessive) intercorrelations between the explanatory variables of mindfulness, stress, SDQ-subscales, impulsivity and sensation seeking (see supplement material 3). We, therefore, explored associations between outcomes with each explanatory factor separately in additional analyses (Table 5), only adjusted for sex/gender and age. The findings from these analyses largely confirm but accentuate the associations from the previous analyses regarding most variables (age, sex/gender, migration, psychological stress, emotional problems, hyperactivity symptoms, impulsivity and sensation seeking). In the additional models, mindfulness was associated with all outcomes, while it was only a significant factor for the unspecified SUD outcome and alcohol dependence in the previous analyses. The odds ratios for mindfulness and impulsivity increased substantially across the assessed outcomes, which may indicate shared variance among the explanatory variables in the previous multivariate regression model.

**Table 5:** Sociodemographic and psychosocial factors and associated likelihood with fulfillment of diagnostic criteria for DSM-IV substance use disorder (*N* = 3999)

	Unspecified SUD	Multiple SUDs	Cigarette dependence	Alcohol abuse	Alcohol dependence	Cannabis abuse	Cannabis dependence
Sex/gender	1.30** **[1.05, 1.61]**	1.40 [0.99, 1.96]	1.52 [0.93, 2.50]	1.37** **[1.08, 1.72]**	1.01 [0.70, 1.50]	1.48 [0.98, 2.23]	1.53 [0.74, 3.18]
Age	2.24*** **[2.05, 2.44]**	2.17*** **[1.88, 2.50]**	2.08*** **[1.69, 2.57]**	2.06*** **[1.88, 2.25]**	2.20*** **[1.88, 2.59]**	2.25*** **[1.88, 2.70]**	2.02*** **[1.48, 2.74]**
Migration	0.92 [0.70, 1.22]	1.01 [0.67,1.52]	1.49 [0.85, 2.62]	0.97 [0.72, 1.31]	0.56* **[0.32, 0.99]**	1.06 [0.63,1.77]	0.86 [0.33, 2.26]
Stress (PSS-4)	1.10*** **[1.05, 1.14]**	1.19*** **[1.12, 1.27]**	1.22*** **[1.12, 1.34]**	1.11*** **[1.06, 1.16]**	1.07* **[1.00, 1.15]**	1.13** **[1.05, 1.21]**	1.40*** **[1.23, 1.59]**
Emotional symptoms (SDQ)	1.13*** **[1.07, 1.18]**	1.24*** **[1.15,1.34]**	1.35*** **[1.21, 1.50]**	1.11*** **[1.06, 1.18]**	1.13** **[1.04, 1.22]**	1.19*** **[1.08, 1.30]**	1.48*** **[1.26, 1.73]**
Hyperactivity-inattention (SDQ)	1.25*** **[1.18, 1.31]**	1.41*** **[1.30,1.53]**	1.46*** **[1.30, 1.63]**	1.24** **[1.17,1.31]**	1.22*** **[1.12, 1.33]**	1.33** **[1.21, 1.47]**	1.48*** **[1.25, 1.75]**
Trait mindfulness (MAAS)	0.56*** **[0.48, 0.66]**	0.45*** **[0.35, 0.57]**	0.39*** **[0.29, 0.56]**	0.60*** **[0.51, 0.72]**	0.46*** **[0.35, 0.61]**	0.55*** **[0.41, 0.74]**	0.36*** **[0.22, 0.59]**
Sensation seeking (SURPS)	1.51*** **[1.32, 1.73]**	1.55*** **[1.25, 1.93]**	1.28 [0.95, 1.74]	1.62*** **[1.40, 1.88]**	1.47** **[1.16, 1.86]**	1.32* **[1.02, 1.71]**	1.02 [0.66, 1.58]
Impulsivity (SURPS)	1.78*** **[1.51, 2.09]**	2.34*** **[1.82, 3.00]**	2.93*** **[2.07, 4.17]**	1.71*** **[1.44, 2.03]**	2.12** **[1.61, 2.78]**	1.58** **[1.17,2.13]**	1.93* **[1.15, 3.22]**

This table depicts the association between DSM-IV substance use disorders (any SUD and subtypes) and each explanatory variable separately but adjusted for demographic characteristics (sex/gender and age). Bold value indicates that odds ratios are statistically significant (*p < 0.05; **p < 0.01; ***p < 0.001). Adjusted Odds Ratios (aORs) and 95% Confidence Intervals (95%-CIs) are based on binary logistic regression models adjusted for sex/gender and age. Sex/gender coding: 1 = male, 0 = female; Migration coding: 0 = no, 1 = yes. Age in years (12–18)

## Discussion

The present study examined prevalence rates of substances use disorders in a large representative sample of German adolescents and associations with mindfulness and related constructs such as impulsivity and sensation seeking. The results add to an empirical base for estimation of treatment needs in the youth population and fill an important gap in the epidemiology of SUDs in Germany. Furthermore, the study contributes to the growing literature on mindfulness as a relevant psychological target for treatment and prevention of substance-related problems.

### Prevalence rates of substance use disorders

The central study results on SUD prevalences indicate that these disorders are relevant in the youth population and treatment needs for this age group may be similar to those in the adult population in Germany [8]. With the exception of alcohol dependence, all SUD subtypes were more prevalent for male compared to female youth and there was consistent increase with age (except for e-cigarettes dependence). The increases in prevalence rates from early to late adolescence are consistent with previous findings [43, 44] and largely match a “natural” life-course pattern of substance use behavior [45, 46].

### Alcohol use disorders

In our adolescent sample, the highest prevalence rate was found for alcohol abuse. Compared to existing recent data from the adult population in Germany, this value is high and even exceeds the prevalence rate of the adult subsample with the highest prevalence rate (age group of 18 to 20 years: 6.7%) [8]. It also clearly exceeds youth prevalence rates for alcohol-related disorders from US-based studies with a range of 1.7% [32] to 4.7% [14]. In turn, the value from our study comes close to the prevalence rate for alcohol abuse from the previous ESDP-study (9.7% [11]). This comparison indicates that although youth drinking in Germany and Europe have been decreasing [47], a substantial proportion of school-aged youths continuous to drink alcohol at levels associated with serious risk of harm. For example, adolescents aged 15 to under 20 are the largest group for emergency care due to acute alcohol intoxication in German hospitals [48].

The diagnostic criteria for alcohol abuse were met more often than those for alcohol dependence (3.1%) and this value is also lower compared to the prevalence reported for the adult population in Germany (4.8%) [8]. This is not surprising given that youth drinking is typically characterized by episodic excessive use (“binge drinking”) [2, 49] and dependence symptoms such as tolerance and withdrawal are less pronounced in adolescence. Given that the neurobiological and pharmacological adaptation processes required for the formation of these physical symptoms usually only occur after prolonged heavy use, their appropriateness as diagnostic criteria for SUDs have been repeatedly criticized from a child and adolescent psychiatric perspective [44, 50, 51].

### Cannabis use disorders

With regard to cannabis use disorders the prevalence rates were much lower than the prevalence rates for alcohol. However, this is not surprising. Cannabis is an illegal substance and less available than alcohol in Germany, which is among the countries with the highest per capita consumption of alcohol in the world [52]. Moreover, this finding matches previous results showing that cannabis use disorders are about a factor of 3–5 times less prevalent than alcohol use disorders in Germany [8, 31]. The distribution also reflects non-clinical patterns of use found in this study and as well as in the general population: while 43.6% of adolescents in this sample have used alcohol at least once in their lives, this proportion was only 8.9% for cannabis (these values are largely similar to those reported in federal statistics [53].

However, the picture is different when the prevalence rates are considered only for those adolescents who have actually used the substance within the past 12 months. For this subsample, prevalence rates were much higher for cannabis compared to alcohol use disorders. This suggests that cannabis users are more likely to meet diagnostic criteria for a SUD than alcohol users. This notion corresponds to previous findings indicating that progression from first use towards problematic use is usually faster for cannabis compared to alcohol use [54].

### Tobacco and e-cigarette dependence

The prevalence for tobacco cigarette dependence outweighed the prevalence of e-cigarette dependence by far. Moreover, while a comparatively small proportion of past-year e-cigarette users met the diagnostic criteria for dependence, this proportion was about 8 times as large for tobacco cigarettes.

In Germany the spreading of e-cigarettes and associated health risks for children and young people have been of recent concern [55]. Internationally, there has been an increase in e-cigarette use during the past years with evidence indicating that the use of e-cigarettes is associated with increased risks for subsequent initiation of tobacco cigarette use [56, 57]. However, studies comparing the amount dependent users of e-cigarettes vs. cigarettes are missing. Part of the problem in comparing the addictive potentials is that e-cigarettes are not a uniform product and different products with varying doses of nicotine are available (see [58] for an overview).

In our survey, the assessment referred to the use of any form of electronic cigarettes; consumption of different products such as tobacco heaters, e-pipes or “e-hookahs” were not examined. Although this assessment appears rather broad, the present findings nevertheless confirm recent results from France [59] indicating that tobacco use continues to be more problematic than e-cigarette use among adolescents in Europe.

### Associations with mindfulness and related factors

Overall, the observed associations largely match with previous findings and added to the explanation of variance in most of the assessed subtypes of SUDs; however, there were similarities and differences between these outcomes. With regard to mindfulness, our findings are comparable in direction and magnitude to meta-analytic associations with use of cigarettes, alcohol and cannabis in population-based samples of adolescents and young adults [27, 60].

Our findings also largely confirm previous studies regarding the role of impulsivity, a multifaceted and central construct in the context of addictive behavior [16]. For our survey-type study, brief assessments of impulsivity and sensation seeking were found to be independently associated with increased risk for SUD of various subtypes. These findings add to existing evidence indicating that both factors contribute independently to risks for substance misuse and can be useful as screening instruments for identifying substance-related problems among adolescents [40, 61]. It should be noted that in our multivariable analyses, mindfulness, impulsivity and sensation seeking as well as dimensions of externalizing (hyperactivity) and internalizing (emotional) problems were correlated but added unique proportions of explained variance for SUD. However, this pattern did not generalize across all different SUD subtypes.

In the additional analyses, the pattern of associations was much more consistent across SUD subtypes and the associations were also typically stronger when assessed separately. For example, stress was substantially correlated with SDQ-dimensions of psychopathology, mindfulness and impulsivity. While stress was not significant in the multivariate models for all outcomes except of cannabis abuse, it was significantly associated across all outcomes in the additional analyses. The associations between impulsivity (to a lesser degree also sensation seeking) and particularly mindfulness were much more consistent and stronger when separate variable associations were assessed.

While multicollinearity between explanatory variables can be problematic in multivariable analyses, the apparent intercorrelations in the present results appear conceptually plausible. In general, SUDs are highly comorbid with other mental problems and share a variety of risk and resilience factors in the spectrum of internalizing and externalizing problems [62, 63]. With regard to mindfulness, impulsivity and emotional and behavioral problems in particular a possible overlap is supported by previous studies [64, 65]. Single and colleagues [28], for example, found that an association between mindfulness and alcohol-related problems was explained by low levels of emotional psychopathology. Other recent research found that dimensions of impulsivity and self-regulation are associated with mindfulness [64]. While in the present study, we explored direct associations of these factors with substance use disorders, the relations between characteristics of mindfulness, impulsivity, sensation seeking, emotional and behavioral problems may include indirect chains of associations and should be examined in more detail in further analyses [65].

### Implications and added value of the study

The present study provides a much needed empirical base for estimating treatment needs for the youth population in Germany [50], which have been derived rather indirectly from inadequate data bases. The methodology is largely comparable to existing survey-based research in representative samples from the general population and provides high levels of generalizability. Samples relying on adolescents in school or in treatment may underestimate substance use because they do not include adolescents who have dropped out of school or are in residential care.

The prevalence rates found in this study provide a baseline for future epidemiological studies. Given that patterns of substance use change historically, it is important to monitor trends and changes in prevalence rates to estimate public health demands.

Interestingly, while youth alcohol use in Germany has been decreasing steadily in the past two decades, the proportion of persons who consume alcohol (and other substances) problematically has been rather stable [11, 31]. This observation may inform public health initiatives towards a stronger focus on targeted intervention approaches for those who are at risk to develop substance use disorders. Currently, prevention programs in the context of substance use focus on universal and mostly school-based measures in the general population [66].

Considering the ongoing initiative to legalize recreational use of cannabis for adults in Germany [67], our data may provide a starting point from which future developments in the extent and prevalence of cannabis use disorders can be evaluated. However, it is clear that long-term longitudinal studies are needed for identifying the developmental conditions that play a role for stability and change of substance (ab) use and dependence. Without longitudinal data, we cannot determine the reliability and validity of the questionnaire-based operationalization of substance abuse and dependence criteria. Likewise, it may be possible that social desirability can influence response behavior for cannabis use, since this substance is illegal in Germany.

Moreover, while our DSM-IV-based prevalence rates allow for a high comparability with previous studies [8, 11, 14, 31], they may underestimate the prevalence in comparison with prospective DSM-5-based studies, which apply a lower diagnostic threshold (for a mild disorder). At least this is suggested by recent survey data from the United States that allows for a direct comparison: the prevalence rates for alcohol and cannabis use disorder (aggregated DSM-IV abuse and/or dependence) in the 12–17 age group were 1.7 and 1.8% in the 2019 survey [32] but 2.8 and 4.1% for at least a mild alcohol and cannabis use disorder based on DSM-5 in the 2020 survey [13].

The distinction of two well-defined categorical clinical entities is similar to the ICD-10 and ICD-11 [3] which is applied in the care systems of most European countries, including Germany. However, statements about the diagnostic compatibility with the classification standards of the ICD (ICD-10 and ICD-11) can only be made with caution because of limited availability of data from youth populations and results from adult populations are mixed. Several studies using adult samples suggest a relatively high level of concordance between ICD-10- and DSM-IV-based prevalence rates. In an Australian study [68], there was a high concordance between both classification systems for alcohol and cannabis dependence which was also higher than the compatibility between both DSM versions. Likewise, a large-scale study of over 12,000 adults from general populations in several countries showed very high overlap between ICD-11 dependence diagnoses for alcohol and cannabis with both ICD-10, as well as DSM-IV and (albeit with cutbacks) DSM-5 [69].

Our results show that higher levels of trait mindfulness are associated with a lower likelihood to meet DSM-IV criteria for SUDs. Likewise, we found an inverse relationship of trait mindfulness with other related risk factors for substance use (disorders), such as impulsivity, stress, and symptoms of psychopathology. Given the high comorbidity with emotional and behavioral problems that is frequently associated with adolescent substance use and the transdiagnostic qualities of mindfulness-based interventions [25, 70], such interventions may be promising for youth populations.

### Limitations

The generalizability of the study results may be limited because of two issues. First, we lack information about those adolescents who did not participate in the survey. We can, therefore, not rule out a possible selection bias and limitations to sample representativeness. Second, the survey was conducted during the Corona pandemic. At the time of data collection, German school-aged children and adolescents were clearly affected by the restrictions to their usual lives although the estimated turning point in the pandemic and related problems across the federal states in Germany was already passed for about six months [71]. Research on the implications of the COVID-19 pandemic related conditions such as the implementation of social distancing measures on adolescent substance use are mixed: a study from Canada shows that the percentage of young alcohol and cannabis users decreased but the frequency of use of both substances increased. In a cohort-study among ninth- and tenth-grade students in Northern California [72] in contrast found that the overall prevalence of e-cigarette, cannabis, or alcohol use did not meaningfully change with a state-wide stay-at-home order.

Comparable data in youth samples in Germany are not available, but there were increases in psychological stress [38] and SDQ-based mental health problems [73] during COVID-19 lockdown in Germany. Although SDQ-scores and stress were included in the statistical analysis and DSM-IV diagnostic criteria refer to a 12 months’ time frame with the cross-sectional study we are unable to determine whether the burden related to COVID-19 has affected the results.

Finally, it should be noted that the psychometric validity of several measures in this survey study was not optimal. The associations found in this study between the various substance use disorders with stress, impulsivity, sensation seeking, as well as the SDQ-dimensions emotional problems and hyperactivity, although theoretically plausible, should be viewed with some caution.

## Conclusion

Our findings indicated that the criterion-based prevalence of substance use disorders and particularly alcohol-related disorders are widespread in a German youth population and exceed the prevalence previously reported in studies from the United States. The results indicated some similarities but also differences to previous studies the general German population. As in previous studies, alcohol use disorders were most prevalent, which can be expected in a high-consumption country for alcohol such as Germany. Among youth who reported actual use within the past year of the assessed substances, prevalence rates were substantially higher for cannabis use disorder compared to alcohol use disorder. The proportion of adolescents who met the criteria for cigarette dependence was many times higher than for e-cigarettes, which played a relatively minor role in the present study. This was similar to rates reported by previous studies in adult populations. Several factors were associated with substance use disorders, such as sex/gender, age, symptoms of internalizing and externalizing problems, mindfulness, sensation seeking and impulsivity. Patterns of associations suggest that these factors may not be entirely independent which confirms previous conceptual and empirical research. These findings contribute to a knowledge base indicating that mindfulness and related factors such as impulsivity may be useful for prevention and intervention for substance use disorders and related behavioral and emotional problems.

## Supplementary Information

Below is the link to the electronic supplementary material.

Supplementary file1 (DOCX 108 KB)

## Data Availability

The data that support the findings of this study are available from the corresponding author upon reasonable request.
