# Supplementary material for: Prevalence of substance use disorders and associations with mindfulness, impulsive personality traits and psychopathological symptoms in a representative sample of adolescents in Germany
Source: Eur Child Adolesc Psychiatry. 2023 Feb 28;33(2):451–65. doi: 10.1007/s00787-023-02173-0 (PMC9972301; doi:10.1007/s00787-023-02173-0)

**Supplement material 1a and 1b:**

| **Supplement Table 1a.** Diagnostic criteria for DSM-IV substance abuse | |
| --- | --- |
| A1. | Continued use despite social or interpersonal problems caused or exacerbated by the effects of the substance |
| A2. | Repeated use of substance in situations in which it is physically hazardous |
| A3. | Repeated use of substance that results in failure to fulfil major role obligations at home, school or work |
| A4. | Repeated substance-related legal problems |
| Diagnosis is met if at least one of the four abuse criteria are experienced within a 12-months period, but the person is not meeting the criteria for a diagnosis of dependence. | |

| **Supplement Table 1b.** Diagnostic criteria for DSM-IV substance dependence | |
| --- | --- |
| D1. | Marked tolerance to a substance |
| D2. | Withdrawal symptoms or the use of that substance to relieve withdrawal |
| D3. | Using more of a substance or using a substance over a longer period of time than intended |
| D4. | An impaired capacity to control use of the substance |
| D5. | Spending a great deal of time obtaining, using, or getting over the effects of a substance |
| D6. | Neglect of alternate activities in order to get or use a substance |
| D7. | Continuing to use a substance despite knowledge of physical or psychological problems due to the use of the substance |
| Diagnosis is met if at least three of the seven dependence criteria are experienced within a 12-months period. | |
| **Note**. In the survey, questions were designed to represent symptom criteria for each of the assessed substances separately. For alcohol and cannabis, a diagnosis of dependence pre-empted that person to be scored with abuse. For cigarettes DSM-IV does not specify criteria for abuse. Criteria for e-cigarettes dependence were adapted from the criteria for nicotine dependence. | |

| **Supplement Table 2.** Prevalence of lifetime-, 12-months- and 30-days substance use (N=4001) | | | | | | | |
| --- | --- | --- | --- | --- | --- | --- | --- |
| **Variable** | | **Cigarettes** | **E-Cigarettes** | **Alcohol** | **Cannabis** | **Any substance use^a^** | **Multiple consumptions^b^** |
|  | | **% (SE)** | **% (SE)** | **% (SE)** | **% (SE)** | **% (SE)** | **% (SE)** |
| **Lifetime use (Total)** | | 14.2 (.006) | 12.1 (.005) | 43.6 (.008) | 8.9 (.005) | 45.7 (.008) | 19.1 (.006) |
|  | **Age^c^** |  |  |  |  |  |  |
|  | 12-13 years | 2.1 (.004) | 2.3 (.004) | 5.4 (.006) | 0.3 (.002) | 7.4 (.007) | 2.0 (.004) |
|  | 14-15 years | 8.9 (.008)*** | 9.3 (.008)*** | 39.9 (.013)*** | 3.6 (.005)*** | 42.0 (.013)*** | 12.3 (.008)*** |
|  | 16-18 years | 30.9 (.013)*** | 24.2 (.012)*** | 83.2 (.010)*** | 22.3 (.011)*** | 85.3 (.010)*** | 42.0 (.013)*** |
|  | **Sex/gender** |  |  |  |  |  |  |
|  | Female | 13.0 (.008)* | 9.5 (.007)*** | 43.5 (.011) | 7.8 (.007)* | 45.3 (.011) | 17.0 (.008)** |
|  | Male | 15.3 (.008) | 14.6 (.008) | 43.8 (.011) | 10.0 (.007) | 46.1 (.011) | 21.1 (.009) |
|  | **Migration** |  |  |  |  |  |  |
|  | Yes | 13.9 (.013) | 12.6 (.012) | 33.7 (.017)*** | 8.1 (.010) | 37.0 (.017)*** | 17.1 (.014) |
|  | No | 14.3 (.006) | 12.0 (.006) | 46.0 (.009) | 9.1 (.005) | 47.8 (.009) | 19.6 (.007) |
| **12-month use (Total)** | | 9.6 (.02) | 6.2 (.023) | 39.8 (.007) | 6.6 (.023) | 41.1 (.008) | 3.3 (.003) |
|  | **Age** |  |  |  |  |  |  |
|  | 12-13 years | 1.0 (.098) | 1.1 (.094) | 3.5 (.058) | 0.1 (.25) | 4.2 (.006) | 0.2 (.001) |
|  | 14-15 years | 5.9 (.043) | 5.4 (.054) | 35.4 (.014)*** | 2.6 (.064) | 36.9 (.013)*** | 1.5 (.003)*** |
|  | 16-18 years | 21.3 (.023) | 11.8 (.028) | 78.2 (.007)*** | 16.7 (.025) | 80.0 (.010)*** | 8.2 (.007)*** |
|  | **Sex/gender** |  |  |  |  |  |  |
|  | Female | 8.9 (.029) | 4.9 (.050) | 39.8 (.010) | 5.9 (.031) | 40.9 (.011) | 2.8 (.003) |
|  | Male | 10.3 (.027) | 7.5 (.029) | 39.9 (.010) | 7.3 (.031) | 41.1 (.011) | 3.8 (.004) |
|  | **Migration** |  |  |  |  |  |  |
|  | Yes | 9.1 (.046) | 5.9 (.051) | 29.9 (.020) | 5.7 (.058) | 32.2 (.017)*** | 3.6 (.007) |
|  | No | 9.7 (.022) | 6.3 (.025) | 42.2 (.007) | 6.8 (.025) | 43.3 (.009) | 3.3 (.003) |
| **30-days use (Total)** | | 5.5 (.025) | 2.0 (.03) | 25.4 (.012) | 2.8 (.03) | 26.9 (.007) | 1.2 (.002) |
|  | **Age** | 105 (5.3) | 36 (1.8) | 493 (25.1) | 41 (2.1) |  |  |
|  | 12-13 years | 0.6 (.144) | 0.3 (.125) | 1.3 (.073) | 0.0 (.00) | 1.7 (.004) | 0.1 (.000) |
|  | 14-15 years | 3.0 (.056) | 1.6 (.053) | 16.8 (.023) | 1.1 (.083) | 18.1 (.010)*** | 0.8 (.002)** |
|  | 16-18 years | 12.7 (.029) | 4.1 (.038) | 56.6 (.014)*** | 7.1 (.033) | 59.3 (.013)*** | 2.7 (.004)*** |
|  | **Sex/gender** |  |  |  |  |  |  |
|  | Female | 5.3 (.037) | 1.8 (.050) | 25.1 (.017) | 2.1 (.045)* | 26.7 (.010) | 0.9 (.002) |
|  | Male | 5.7 (.034 ) | 7.5 (.037) | 25.7 (.017) | 3.5 (.041) | 27.1 (.010) | 1.5 (.003) |
|  | **Migration** |  |  |  |  |  |  |
|  | Yes | 4.8 (.060) | 2.0 (.071) | 18.6 (.032) | 2.3 (.075) | 19.9 (.014)*** | 1.2 (.004) |
|  | No | 5.7 (.028) | 2.1 (.033) | 27.0 (.013) | 2.9 (.033) | 28.5 (.008) | 1.2 (.001) |
| **Note.** Results based on weighted dataset. **^a^** consumption of at least one substance; **^b^** consumption of at least two substances. **^c^** comparisons (*Χ*^2^-tests) between youngest age group (12-13 years) with middle age group (14-15 years) and middle age group with oldest age group (16-18 years). *p <.05, **p<.01, ***p<.001. | | | | | | | |

**Supplement material 3**:

| **Supplement Table 3**. Intercorrelations among study variables (N=4001) | | | | | | | | | | | | | | | | | |
| --- | --- | --- | --- | --- | --- | --- | --- | --- | --- | --- | --- | --- | --- | --- | --- | --- | --- |
| Variable | | 1 | 2 | 3 | 4 | 5 | 6 | 7 | 8 | 9 | 10 | 11 | 12 | 13 | 14 | 15 | 16 |
| 1 | Age |  |  |  |  |  |  |  |  |  |  |  |  |  |  |  |  |
| 2 | Sex/gender | .00 |  |  |  |  |  |  |  |  |  |  |  |  |  |  |  |
| 3 | Migration | -.03 | -.02 |  |  |  |  |  |  |  |  |  |  |  |  |  |  |
| 4 | Unspecified  SUD | .33*** | -.04* | -.02 |  |  |  |  |  |  |  |  |  |  |  |  |  |
| 5 | Multiple  SUDs | .20*** | -.03 | -.01 | .56*** |  |  |  |  |  |  |  |  |  |  |  |  |
| 6 | Cigarette  dependence | .13*** | -.03 | .02 | .37*** | .50*** |  |  |  |  |  |  |  |  |  |  |  |
| 7 | Alcohol  abuse | .28*** | -.04** | -.01 | .88*** | .57*** | .27*** |  |  |  |  |  |  |  |  |  |  |
| 8 | Alcohol  dependence | .18*** | .00 | -.04* | .50*** | .60*** | .23*** | .33*** |  |  |  |  |  |  |  |  |  |
| 9 | Cannabis  abuse | .17*** | -.03 | -.01 | .45*** | .61*** | .24*** | .34*** | .26*** |  |  |  |  |  |  |  |  |
| 10 | Cannabis  dependence | .09*** | -.02 | -.01 | .25*** | .43*** | .32*** | .22*** | .22*** | .46*** |  |  |  |  |  |  |  |
| 11 | Stress  (PSS-4) | -.06*** | .20*** | .06*** | .08*** | .09*** | .07*** | .08*** | .04** | .05*** | .08*** |  |  |  |  |  |  |
| 12 | Emotional  problems (SDQ) | .14*** | .36*** | .03 | .09*** | .09*** | .09*** | .08*** | .06*** | .06*** | .08*** | .52*** |  |  |  |  |  |
| 13 | Hyperactivity-inattention (SDQ) | -.08*** | -.03 | .05** | .10*** | .12*** | .10*** | .10*** | .06*** | .09*** | .07*** | .30*** | .24*** |  |  |  |  |
| 14 | Trait Mindfulness  (MAAS-A) | -.14*** | -.18*** | -.06*** | -.13*** | -.11*** | -.09*** | -.11*** | -.11*** | -.07*** | -.07*** | -.46*** | -.49*** | -.38*** |  |  |  |
| 15 | Sensation Seeking  (SURPS) | .09*** | .00 | -.03* | .12*** | .08*** | .04* | .12*** | .07*** | .05** | .11 | .03 | -.09*** | .07*** | -.07*** |  |  |
| 16 | Impulsivity  (SURPS) | -.04** | .04* | -.05*** | .08*** | .09*** | .09*** | .08*** | .07*** | .04* | .03* | .31*** | .25*** | .39*** | -.42*** | .09*** |  |
| **Note.** Correlations based on weighted dataset. SUD diagnosis: endorsement of any of the assessed substance use disorders; Dummy coded variables: sex/gender (female=0; male=1), Migration (yes=1; no = 0). PSS-4: Perceives Stress Scale 4; SDQ: Strengths and Difficulties Questionnaire; MAAS-A: Mindful Awareness and Attention Scale- Adolescent; SURPS: Substance Use Risk Profile Scale.  *p<.05; **p<.01; ***p<.001 | | | | | | | | | | | | | | | | | |


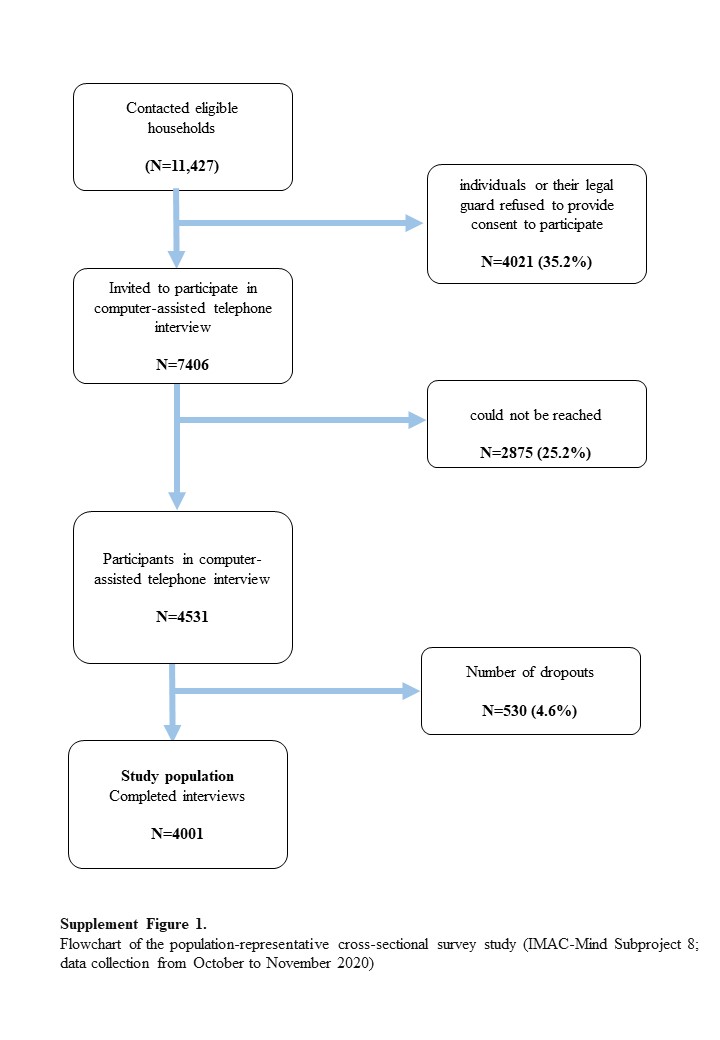

Supplement: Supplementary file 1 — Supplementary file1 (DOCX 108 KB) [file 787_2023_2173_MOESM1_ESM.docx]
